# Translation, cross-cultural adaptation and psychometric properties of the Nepali versions of numerical pain rating scale and global rating of change

**DOI:** 10.1186/s12955-017-0812-8

**Published:** 2017-12-04

**Authors:** Saurab Sharma, Joshna Palanchoke, Darren Reed, J. Haxby Abbott

**Affiliations:** 1 Department of Physiotherapy, Kathmandu University School of Medical Sciences, Dhulikhel, Kavre Nepal; 2 Centre for Musculoskeletal Outcomes Research, Dunedin School of Medicine, University of Otago, Dunedin, New Zealand; 3 Scheer Memorial Hospital, Banepa, Nepal; 4 University of Sydney, Sydney, Australia

**Keywords:** Outcome measure, Assessment, Pain, Global change, Pain assessment, Numerical rating scale, Pain intensity, Pain measurement, Outcome measurement, Global impression of change

## Abstract

**Background:**

Pain intensity and patients’ impression of global improvement are widely used patient-reported outcome measures (PROMs) in clinical practice and research. They are commonly assessed using the Numerical Pain Rating Scale (NPRS) and Global Rating of Change (GROC) questionnaires. The GROC is essential as an anchor for evaluating the psychometric properties of PROMs. Both of these PROMs are translated to many languages and have shown excellent psychometric properties. Their availability in Nepali would facilitate pain research and cross-cultural comparison of research findings. Therefore, the objectives of this study were to translate and cross-culturally adapt the NPRS and GROC into Nepali and to assess the psychometric properties of the Nepali version of the NPRS (NPRS-NP).

**Methods:**

After translating and cross-culturally adapting the NPRS and GROC into Nepali using recommended guidelines, NPRS-NP was administered to 104 individuals with musculoskeletal pain twice. The Nepali version of the GROC (GROC-NP) was administered at the follow-up for anchor-based assessment. (1) Test-retest reliability and minimum detectable change (MDC) among the stable group, (2) construct validity (by single sample t-test within the improved group and independent sample t-test between groups), and (3) concurrent validity were assessed. Receiver operating characteristic (ROC) curves were plotted to determine the responsiveness of the NPRS-NP using the area under the curve (AUC), and minimum important changes (MIC) for small, medium and large improvements.

**Results:**

Significant cultural adaptations were required to obtain relevant Nepali versions of both the NPRS and GROC. The NPRS-NP showed excellent test-retest reliability and a MDC of 1.13 points. NPRS-NP demonstrated a good construct validity by significant within-group difference in mean of NPRS score- *t(63)= 7.57*, *P* < 0.001 and statistically significant difference of mean score- *t*(98)= -4.24, *P* < .001 between the stable and improved groups. It demonstrated moderate concurrent correlation with the GROC-NP; *r* = 0.43, *P* < 0.01. Responsiveness of the NPRS-NP was shown at three levels with AUC = 0.68–0.82, and MIC = 1.17–1.33.

**Conclusions:**

The NPRS and GROC were successfully translated and culturally adapted into Nepali. The NPRS-NP demonstrated good reliability, validity and responsiveness in assessing musculoskeletal pain intensity in a Nepali population.

**Electronic supplementary material:**

The online version of this article (10.1186/s12955-017-0812-8) contains supplementary material, which is available to authorized users.

## Background

Outcome measurement is essential to monitoring and improving the quality and effectiveness of health care [1]. Assessment of pain intensity [2] and patients’ impression of global improvement [3] are important “patient-centred” outcomes in both clinical practice and research, as patients are asked to rate their own pain intensity and global change in their health status [4, 5]. Further, assessment of patients’ impression of global improvement is recommended as an anchor for assessment of the measurement properties of patient-reported outcome measures (PROMs) [6].

Pain intensity is often the primary focus of treatment [7], and is a preferred outcome of assessment in both clinical practice and research for conditions such as cancer, rheumatic diseases, low back/ neck conditions and post-operatively [8–11]. Pain intensity is routinely assessed in clinical practice using the Numerical Pain Rating Scale (NPRS) [12]. It has acceptable psychometric properties. Out of many versions of numerical rating scales, the 11-point NPRS is commonly preferred [4, 9]. The anchor at the left is 0, corresponding to “no pain”, and the anchor at the right side means “worst possible pain” or “maximum pain”. The NPRS is a very simple to use measure, can be administered by patient self-report, or verbally by face-to-face interview, or over a telephone, and has wide applicability to a variety of pain-related conditions [9, 13–15]. One of the advantages of this measure is that it can also be used in individuals with low literacy. It is used routinely in many countries and languages [9].

The global rating of change (GROC) scale was designed for use as an external anchor to determine minimal important change of health-related quality of life measures [16]. The GROC scale is easy to administer, requires minimal skills or training, has good reproducibility, and is sensitive to change [1, 17]. While scores correlate with pain, disability and quality-of-life measures, the open nature of the question allows the patient to take into account other factors that he or she may consider important in his or her clinical situation [3]. It is a Likert scale with a mid-point representing “no change”, a left anchor representing “very much worse” and a right anchor representing “very much better” or “recovered completely”. A variety of GROC scales have been used in research including 15 points, 11 points and 7 points [3]. The originally proposed scale was the 15-point scale [16], while in contemporary use 11-point and 7-point measures are recommended [3].

Use of outcome measures is limited in Nepal because of low literacy levels, unavailability of measures in Nepali and unawareness of need and usefulness of outcome measures. Despite the acceptable validity and reliability and wide applicability of NPRS and GROC measures, neither the NPRS nor GROC are available in Nepali. Before PROMs can be used in clinical practice and research, they should be translated, cross-culturally adapted and validated in the language of the target population [18]. For a measure to be acceptable to use, it is important to know its measurement properties such as reproducibility, validity and responsiveness to change due to treatment or time [18, 19]. Translation of these measures to Nepali using standard recommended guidelines can improve their wide use in both research and patient care in Nepal. Translation of GROC is particularly important to provide an external anchor that researchers in future can use to investigate the clinimetrics of other outcome measures in Nepal.

Therefore, the primary aim of this study was to translate and cross-culturally adapt the NPRS and GROC in accordance with internationally accepted guidelines [18]. Secondary objectives of the study were to evaluate, using a GROC anchor-based approach, the psychometric properties of the Nepali version of the NPRS (NPRS-NP) including the: test-retest reliability, minimum detectable change (MDC), construct and concurrent validity, and the minimum important change (MIC). We hypothesized that translation of the NPRS and GROC to Nepali will provide outcome measure instruments with acceptable psychometric properties.

## Methods

The study protocol was approved by Institutional Review Committee of Kathmandu University School of Medical Sciences, Dhulikhel, Nepal, and complies with the principles outlined in the Declaration of Helsinki. Every participant provided a written informed consent prior to the start of the study. In the event participants were unable to sign the consent form themselves, a witness signed for them. The conduct and reporting of this research was guided by the guidelines proposed by Beaton and colleagues in 2000 for the process of cross-cultural adaptation of self-report measures [18] and by the COnsensus-based Standards for the selection of health Measurement INstruments (COSMIN) guidelines [20].

### Participants

To be eligible to participate in the study, participants were required to be: (1) over 18 years, (2) a citizen of Nepal, (3) able to understand and speak Nepali fluently, (4) say numbers from 0 to 10 in order, and (5) currently experiencing musculoskeletal pain. Exclusion criteria included: any past surgeries related to the current pain; recent history of trauma; presence of red flags suggesting the presence of tumor and infection; and diagnosed psychiatric illnesses. A sample more than 100 is considered adequate in order to assess the psychometric properties of a patient-reported outcome measure [20], therefore, we recruited 104 individuals with musculoskeletal pain who consented to participate in the study and completed all the measures. Of these, 75 (72%) were recruited from the Physiotherapy Out-patient Department of Dhulikhel Hospital and 29 (28%) from the surrounding community. This gave a representative mix of rural and semi-urban participants. We recruited participants between October 2015 and April 2016.

The study was conducted in two phases: *Phase 1* - the translation and cross-cultural adaptation of NPRS and GROC to Nepali, including the pre-testing of the translated Nepali versions; and *Phase 2* – investigation of the psychometric properties of NPRS-NP.

### Phase 1: Translation process

The translation of NPRS and GROC into Nepali followed the standard guidelines for translation and cross-cultural adaptation of patient-reported outcome measures [18]. We chose to translate these measures into Nepali because Nepali is the national language of Nepal; it is the most common language spoken in Nepal, with 45% Nepalese speak Nepali as the first language, followed by “Maithili” (12%) [21]; and it is taught in schools as a compulsory subject. The translation process included:

#### Forward translations

Three native Nepali speakers (one physiotherapist, one professional translator and one naïve non-medical professional) independently translated the original English versions of the NPRS and GROC to Nepali, resulting in 3 versions: T_1,_ T_2_ and T_3_.

#### Synthesis

A single Nepali version (T_4_) was created following discussion and consensus among the three translators and the principal investigator (SS).

#### Back-translations

T_4_ was then back-translated independently by three native English speakers unaware of the purpose of the translation and blind to the original English version resulting in 3 versions: T_5_, T_6_ and T_7_. Inconsistencies were discussed among the back translators and a single synthesized version was produced.

#### Expert committee meeting

An expert committee was formed which consisted of the researchers, translators, methodologist, and a language expert (professional translator). Discussions were undertaken to resolve any discrepancies in the translations that did not reflect the original English version. A final Nepali version (T_8_) was approved after significant (cross-cultural) modifications on both the measures (see the Results section below). Questionable words or phrases in the Nepali version were replaced with alternative Nepali wordings which the committee considered to be reasonable cultural adaptations that maintained the meaning of the English version but were not a direct literal translation. In some instances two options were put forward to be evaluated during the pre-testing of the translation process to obtain the most appropriate option. Translators who were not available to attend the meeting in person were contacted to confirm that all parties were in agreement. From these discussions pre-final versions of the NPRS and GROC were created (T_NP_). All translated versions (with the final back translated English version) were then sent to a senior researcher (JHA) for final comments and approval.

#### Pre-testing

The approved T_NP_ versions of NPRS and GROC were then pre-tested on 30 individuals with self-reported musculoskeletal pain. This sample selected was representative of population age, sex and education level. During the pre-testing, participants were interviewed to complete the T_NP_ versions of NPRS and GROC. The participants were asked if they understood the actual meaning of the T_NP_ upon completion. The participants were also asked for their preference in any unresolved alternative Nepali translations of word choices put forward by the expert committee, and majority preferences were adopted. In response to participants’ feedback, minor corrections were made to improve the sentence structure of the instructions to make it easier for the participants to understand, and the final Nepali versions of NPRS and GROC were finalised (NPRS-NP and GROC-NP respectively).

### Phase 2: NPRS-NP psychometric testing procedure

A longitudinal single-arm cohort design was adopted to assess the test-retest reliability, minimal detectable change (MDC) and minimal important change (MIC) of the NPRS-NP. Data were collected at two time points, at an initial assessment and between 1 and 2 weeks after at a follow-up assessment. No information about the previous NPRS-NP scores were provided to the study participants at the follow-up assessment. The 7 – item Nepali version of GROC (GROC-NP) was also administered independently at the follow-up to assess the participants’ perception of their global rating of change. The research assistant (JP) administering the measures was trained by the principal investigator (SS). All the research participants were interviewed in order to maintain the uniformity of the data collection and not to exclude illiterate participants. To minimize loss to follow-up, phone call interviews were conducted for any participants recruited from the hospital who could not attend subsequent follow-up appointments. To facilitate follow up among the community participants, a research assistant visited individuals at a time convenient to them.

### Data analysis

Data were manually entered into Microsoft Excel and later were transferred to Statistical Package for Social Sciences (SPSS) version 24 for further analysis. Sociodemographic variables including age, sex, ethnicity, education and occupation were reported using descriptive statistics. Distribution of pain was reported as frequency count and percentage by body part affected, and duration of pain (in months) was reported as mean and standard deviation. To differentiate between the responders (Improved group) versus non-responders (Stable group) and to report small, medium, and large improvements (changes) in their NPRS-NP scores, GROC-NP was used as an external anchor [3]. Participants who chose “same as before”, a score of ‘4’ on GROC-NP were classified as the stable or unchanged group, whereas the participants who chose “slight improvement” ‘5’, “moderate improvement” ‘6’ or “a lot of improvement” ‘7’ were classified as responders [22]. Three sensitivity analyses were performed separately on the groups that achieved small, medium and large improvements [12].

For both the initial measurement and final measurement, average scores of NPRS-NP current, minimum, and maximum pain intensities were reported. Change in NPRS-NP scores was computed for individual participants by subtracting the NPRS-NP final measurement from the baseline score.

### Reliability

*Test-retest reliability* was evaluated for the stable group by using Intraclass Correlation Coefficient (ICC). ICC values closer to 1.0 indicate higher test-retest reliability [6]. We hypothesized that the test-retest reliability would be excellent for the stable group which will lie between 0.7–0.9 [23–25].

It has been suggested that ICC scores do not take into account the scale of measurement and the size of error that is clinically relevant [26]. Therefore, a complementary way of measuring reliability or limit of agreement was also performed using ‘Bland-Altman Plots’, where the difference between baseline and final NPRS-NP values (in Y-axis) were plotted against the mean of NPRS-NP scores at baseline and final measurement (in X-axis) [26, 27].

*Minimal detectable change (MDC)* is the lowest estimate of change of an outcome measure beyond random measurement error [1]. MDC_90_ (MDC at the 90% confidence margin) was calculated for the NPRS-NP using the formula, MDC_90_ = z x √2 x SEM, where SEM is the standard error of measurement and z = 1.64 (z score for estimating a 90% confidence interval). We used square root of 2, because a total of two measurements were done for test-retest stability. Finally, we calculated SEM manually by using the formula, SEM = SD (1 - r)^1/2^ [1] where SD is the standard deviation for the mean change of NPRS-NP score from baseline to final measurement, and r = reliability coefficient i.e. Intra-class Correlation Coefficient (ICC) of the stable group. We hypothesized that the MDC_90_ value would lie between 0.5 and 2.5 [25, 28].

### Validity

*The construct validity* of the NPRS-NP was examined in two stages [1]. In the first stage, mean change of NPRS-NP score was tested within the improved group by using a one sample *t*-test. In the second stage, mean change scores were tested between stable and improved groups using independent samples *t-*test. It was hypothesized the NPRS-NP would demonstrate construct validity with a significant difference *P* < 0.05 in the NPRS-NP score within the group that “improved” and in the NPRS-NP scores between the stable group and the improved group.

*The concurrent validity* was evaluated by comparing the difference of NPRS-NP scores at baseline and final measurement with the score of the GROC-NP. We hypothesized that NPRS-NP would moderately (but significantly *P* < 0.05) correlate with GROC-NP score considering Spearman correlation coefficients of 0.36 to 0.67 to be moderate correlation [29].

### Responsiveness

Responsiveness is the validity of an instrument for assessing change over time. Responsiveness was evaluated in five steps as recommended by de Vet and colleagues [6]: (1) GROC-NP was used as the external anchor for the construct of interest (for the assessment of pain intensity using NPRS-NP), (2) individuals with musculoskeletal pain were chosen as the population of interest as they experience varying levels of pain intensity, (3) we considered that the AUC of 0.7 or more acceptable for the ability of NPRS-NP to differentiate between the groups that improved (4) the changes in scores of NPRS-NP over two time points were calculated with the independently collected GROC scores, and (5) accuracy of the classification between changes in NPRS-NP scores and the responder/ stable categories were assessed using a Receiver Operator Characteristic (ROC) curve.

*Area under this curve (AUC)* indicates the accuracy of NPRS-NP for differentiating between the group that improved or remained stable. The value of AUC for the difference of NPRS-NP closer to “1” indicates better agreement with the GROC-NP as an external anchor or the gold standard where AUC = 0.5 means that NPRS-NP cannot accurately differentiate between the group that improves and that does not beyond chance [30, 31]. Sensitivity analyses were conducted, with ROC curves and values of AUC determined for the sub-groups which demonstrated small improvement (GROC = 5), medium improvement (GROC = 6) and large improvement (GROC = 7). It was hypothesized that the AUC values would be equal to or more than 0.7 in each instance.

*Minimal important change (MIC)* for NPRS-NP was identified with reference to the patient reported score of GROC-NP to differentiate the group that improved and that did not, at three levels of meaningful change as described above. Sensitivity and specificity values were also recorded. We hypothesized that NPRS-NP would be sensitive to change with MIC value between 1.1 and 3.5 as reported in the literature [12, 25, 28, 32, 33].

## Results

### Phase 1: Translation and cross-cultural adaptation

NPRS: An important change was made to the right anchor of the T4 version of NPRS-NP during the expert committee meeting. The literal translation of “worst pain possible” or “worst imaginable pain” did not convey the original meaning in the Nepali language, it sounded ‘funny’. The expert committee’s proposal of two alternative anchors were more natural in Nepali and translated back to English as “extreme pain” and “unbearable pain”. Participants in the pre-testing phase when given the choice of the three Nepali end anchor options gave a unanimous preference for the two culturally adapted phrases. Therefore, the Nepali translation of “worst possible pain” was discarded and translations of both “extreme pain” and “intolerable pain” were retained. See Additional file 1 for the final Nepali version of NPRS.

GROC: Initially, translation of the original 15 point GROC [16] was attempted. The expert committee’s discussion however, highlighted that the Nepali translations for each item of GROC were not reflective of the English items as the meaning of the items could not be replaced by Nepali words in the increasing order from 7 to 15 and decreasing order from 7 to 1, there were too many subtle gradations. The committee decided to adopt the 7-point version of the GROC which is a recommended version [3]. The 7-point measure is a numerical rating scale with verbal descriptor for each item with the mid-point “4” which means “no change”, the left anchor “1” means “very much worse” and the right anchor “7” means “recovered completely” or “very much better”. A score more than or equal to 6 on the scale is considered meaningful improvement [3, 34]. The most applicable seven items from the 15-item GROC translations were retained to comprise the 7-item Nepali version of the GROC (GROC-NP) for pre-testing. During the pre-testing, all the participants (*N* = 30) could identify numbers between 0 and 10, and could understand and complete the scale without difficulty. Semantic equivalence of the GROC-NP was assured. Only minor changes were made in the sentence structure of the instruction during the pre-testing after the feedback from the participants. See Additional file 2 for the final Nepali version of GROC.

### Phase 2: Psychometric properties of the NPRS-NP

All of the 104 participants (100%) completed the follow up assessment. The baseline and the final assessments for all the participants were performed with an average interval of 11.5 (SD 3.5) days while the duration ranged from 6 to 18 days.

### Responders versus non-responders

Out of the 104 participants, 62% (*n* = 64) reported >4 on the GROC-NP scale and therefore were classified as the ‘responders’ and considered the “improved group”. Whereas 35% (*n* = 36) reported “no change” (4) on GROC scale and were considered the stable group. Four (4%) reported worsening (GROC < 4).

### Demographic characteristics

Demographic information collected from the participants is presented in Table 1. The majority of the participants were female, 69% (*n* = 72); and half the participants had only attended a primary school or less. More than half of the participants, 56% (*n* = 58) reported an active lifestyle as they either worked at home or on the fields as farmers.

**Table 1:** Description of the participants with scores of numerical pain rating scale and global rating of change

Variables	Frequency (%)	Mean (SD)
Age in years		41.2 (13.5)
Sex		
Male	32 (31%)	
Female	72 (69%)	
Total	104 (100%)	
Ethnicity		
Newar	34 (33%)	
Brahmin	23 (22%)	
Chettri	16 (15%)	
Others	31 (30%)	
Education		
No school	41 (39%)	
Primary	11 (11%)	
Secondary	17 (16%)	
Higher secondary	16 (15%)	
Bachelor and above	19 (18%)	
Occupation		
Agriculture and house work	28 (27%)	
House work only	22 (21%)	
Agriculture only	8 (8%)	
Sitting job (Office/ business)	8 (8%)	
No work	6 (6%)	
Others	32 (31%)	
Site of pain		
Low back pain	48 (46%)	
Knee pain	21 (20%)	
Shoulder pain	13 (13%)	
Neck	9 (9%)	
Elbow pain	5 (5%)	
Others	8 (8%)	
Total duration of pain (in months)		21.7 (34)
Time between evaluations (in days)		11.5 (3.5)
GROC-NP at follow-up		
Worsened (GROC <4)	4 (4%)	
Stable group (GROC = 4)	36 (35%)	
Improved group (GROC = 5–7)	64 (61%)	
Small improvement (GROC = 5)	30 (29%)	
Medium improvement (GROC = 6)	23 (22%)	
Large improvement (GROC = 7)	11 (11%)	
NPRS scores		
NPRS-NP baseline	104	4.27 (1.63)
NPRS-NP follow-up	104	3.36 (1.56)
NPRS-NP change	104	0.90 (1.49)

*Abbreviations*: *GROC-NP* Nepali version of global rating of change, *NPRS-NP* Nepali version of numerical pain rating scale, *SD* standard deviation

### Assessment of pain site and outcomes

Almost half the participants, 46% (*n* = 48) had low back pain (LBP) and 20% (*n* = 22) had knee pain. Table 1 includes other sites of pain.

### Reliability

**Fig. 1:**
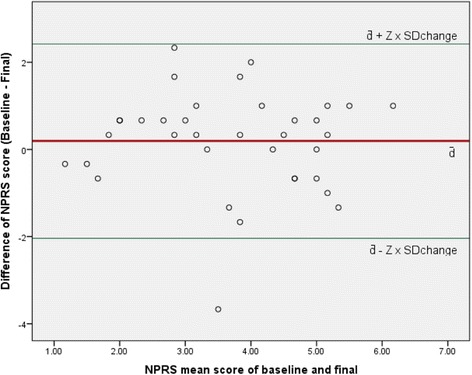
Bland-Altman plot of Numerical Pain Rating Scale.ᅟY-axis is the change of NPRS-NP scores between baseline and follow-up measurements and X-axis is the mean of NPRS-NP scores at the baseline and final measurements. Solid line is the mean change of score (d̄); and green lines are d̄ ± Z x SDchange (where Z = 1.64 for 90% confidence interval)

The ICC statistic for the test-retest reliability of NPRS-NP for the stable group (*n* = 36) at two week follow-up, the SEM, and the MDC_90_ are presented on the Table 2. Bland-Altman Plot drawn between (1) the differences between the NPRS-NP scores in the baseline and final measurements in the Y- axis, and (2) the mean of the two scores in the X-axis is shown in Fig. 1.

**Table 2:** Reliability of Nepali- numerical pain rating scale

NPRS	GROC score	Sample (N)	Test-retest reliability as ICC (95% CI)	SEM	MDC_90_
Stable group	4	36	0.81 (0.63, 0.90)	0.49	1.13

*Abbreviations*: *NPRS* numerical pain rating scale, *GROC* global rating of change, *ICC* intraclass correlation coefficient, *CI* confidence interval, *SEM* standard error of measurement, *MDC*_*90*_ minimum detectable change at 90% CI

### Validity

#### Construct validity

The single sample *t*-test demonstrated a significant difference of mean NPRS-NP scores at baseline and follow-up- *t*(63) = 7.57, *P* < 0.001 in the improved group. The independent sample *t*-test also revealed a significant difference- *t*(98) = −4.24, *P* < 0.001 between the stable and improved group.

#### Concurrent validity

The mean change of the NPRS-NP scores demonstrated significant correlation (*r* = 0.43, *P* < 0.001) with GROC-NP scores for the total sample.

### Responsiveness

**Fig. 2:**
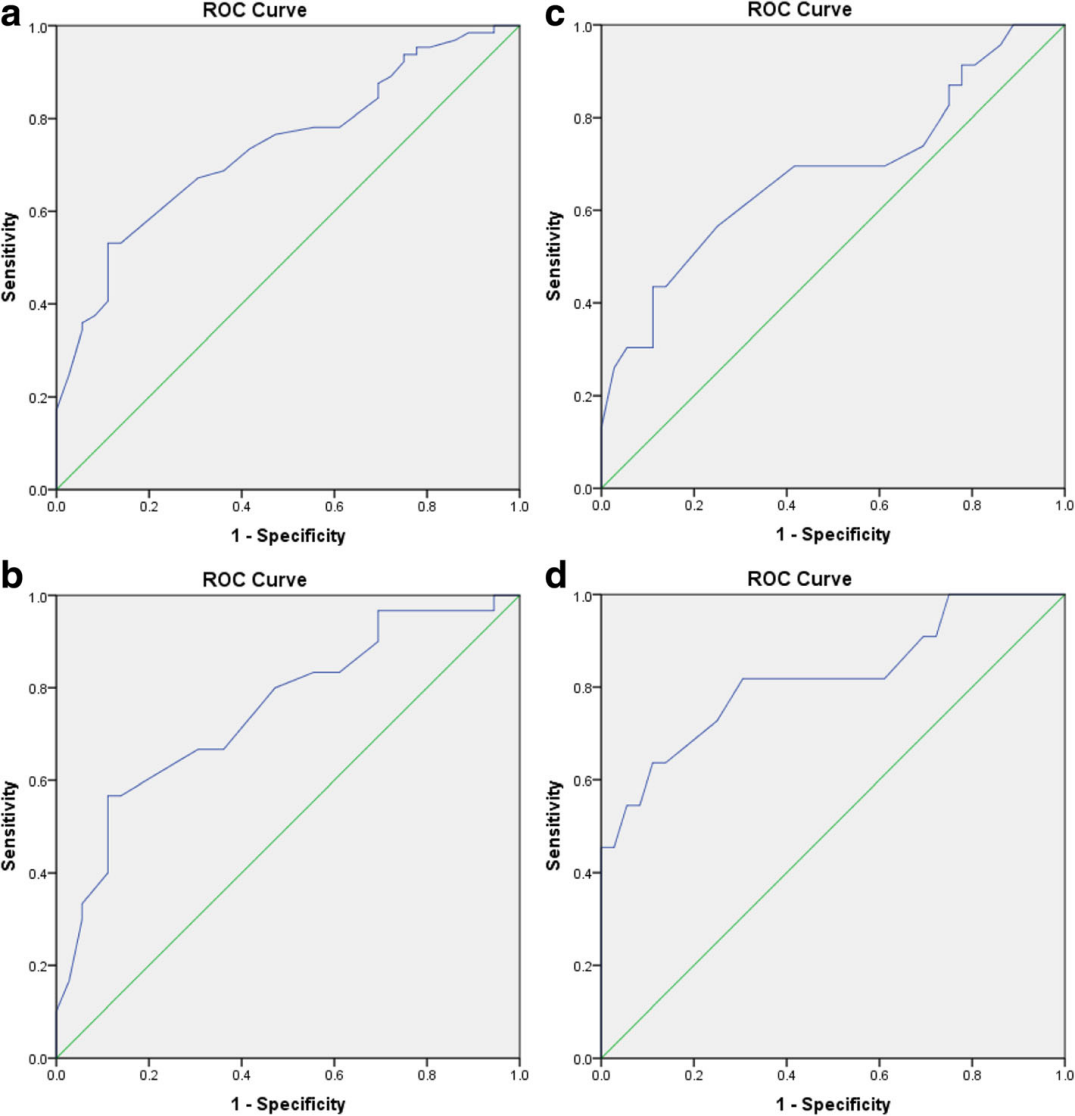
Receiver operating characteristic (ROC) curves.ᅟ**a** Receiver Operating Characteristic (ROC) Curve for the stable group (GROC=4) versus improved group (GROC=5-7). Area under this curve (AUC) indicates the accuracy of NPRS-NP for differentiating between the stable and improved group with the value of AUC closer to “1” indicating better agreement with the GROC-NP as an external anchor. **b** Receiver Operating Characteristic (ROC) Curve for small improvement group (GROC=5). Area under this curve (AUC) indicates the accuracy of NPRS-NP for differentiating between the stable group and the group that had a small improvement with the value of AUC closer to “1” indicating better agreement with the GROC-NP as an external anchor. **c** Receiver Operating Characteristic (ROC) Curve for the medium improvement group (GROC=6). Area under this curve (AUC) indicates the accuracy of NPRS-NP for differentiating between the stable group and the group that had a medium improvement with the value of AUC closer to “1” indicating better agreement with the GROC-NP as an external anchor. **d** Receiver Operating Characteristic (ROC) Curve for the large improvement group (GROC=7). Area under this curve (AUC) indicates the accuracy of NPRS-NP for differentiating between the stable group and the group that had a large improvement with the value of AUC closer to “1” indicating better agreement with the GROC-NP as an external anchor

The ROC curves for the differences of NPRS-NP scores at baseline and final measurements between the improved and stable group are shown in Fig. 2a. Secondary analyses are shown in Fig. 2b–d for the; (1) small improvement group and stable group, (2) medium improvement group and stable group and, (3) large improvement group and stable group. The values of MIC for small and medium improvement was 1.17 and 1.33 for large improvement. The values of AUC, sensitivity and specificity are presented in the Table 3.

**Table 3:** Responsiveness of Nepali- numerical pain rating scale

	AUC			MIC	Sensitivity	Specificity
	AUC	95% CI				
Primary analysis (GROC 4 vs GROC 5–7)	0.74	0.64	0.84	1.17	0.53	0.89
Small improvement (GROC 4 vs GROC = 5)	0.75	0.64	0.87	1.17	0.57	0.89
Medium improvement (GROC 4 vs GROC = 6)	0.68	0.54	0.83	1.17	0.43	0.89
Large improvement (GROC 4 vs GROC = 7)	0.82	0.67	0.98	1.33	0.64	0.89

*Abbreviations*: *AUC* area under the curve, *CI* confidence interval, *MIC* minimum important change, *GROC* global rating of change

## Discussion

We translated NPRS and GROC into Nepali with significant cultural adaptations and that the NPRS-NP demonstrated good to excellent psychometric properties as hypothesized.

### Translation and cross-cultural adaptation

Direct translation of an outcome measure developed for one language or culture to another language may not result in a valid instrument [18, 35]. This study provides clear evidence for the need of cross-cultural adaptation after translation of a measure to the target language. For example, “worst imaginable pain” or “pain as bad as you can imagine” are widely used as the right anchor on a NPRS [9] in many languages, and is recommended by the Initiative on Methods, Measurement, and Pain Assessment in Clinical Trials (IMMPACT) [4]. In the current study, translation of this anchor to Nepali was attempted by three independent translators, however none of the versions sounded “natural”. We proposed alternative Nepali translations that mean “maximum pain” and “intolerable pain” as these phrases as right anchor which are simpler and easily understood. During the pre-testing phase, individuals with musculoskeletal pain were further interviewed and asked for their preference among the three options of the right anchor proposed. None of the participants chose the Nepali translation of “worst imaginable pain”, so it was omitted from final Nepali translation. A previous systematic review reported that both “maximum pain” and “intolerable pain” are used as the right anchor for the NPRS in languages other than Nepali [9].

Further, we encountered difficulties attempting to translate the original 15-item GROC scale developed by Jaeschke and colleagues [16]. The ordinal gradations of the 15-item scale could not be adequately translated, so we produced a 7-item scale in the end. This 7-item scale retained the ordinal property of the scale such that increase of score from 4 to 7 reflect gradual improvement in health status and decrease in score from 4 to 1 reflect worsening of the condition. The 7-item scale is extensively used in research [3, 36]. According to previous research in LBP by Lauridsen and colleagues [36], reduction in the number of items from 15 to 7 does not appear to impact on the performance of the measure. In that study, both the 7-item GROC and 15-item GROC were administered, finding that the classification of improvement did not significantly change by the choice of the GROC scale. They further reported that there were no differences in the performance of the two versions of GROC irrespective of how stringent the criteria was for the improved group. The briefer scale should be easier for the participants to complete because of the lesser number of items. Moreover, the 7-item GROC is the recommended scale to use for chronic pain trials by IMMPACT [4].

### Reliability

The finding of the current study supported our hypothesis that NPRS would demonstrate an excellent test-retest reliability (ICC = 0.81) for the stable group. The test-retest reliability of NPRS-NP is comparable to other studies investigating the clinimetric properties of the NPRS [25, 28], but lower than the 48 h test-retest reliability of the Arabic version (ICC = 0.89) [37]. We followed up participants in the current study after one to two weeks (mean 11.5 days with 3.5 days of SD), as recommended in the literature for the test-retest reliability, which is long enough to avoid recall bias [6]. The duration of the follow-up in our study lies between the duration reported in the previous studies i.e., interval of 2 to 4 days, and between 2 and 4 weeks in different studies [25, 28, 37]. This shows that the reliability of NPRS is similar or comparable irrespective of duration of the follow-up. Similarly, the value of MDC_90_ of NPRS-NP in the current study was 1.13, which was found to be lower than that of the English version (MDC = 2.1 and 2.5) [25, 28], and the Arabic version (MDC = 1.96) [37].

### Validity

As hypothesized, the NPRS-NP demonstrated good construct validity. We found the NPRS-NP demonstrated a significantly different scores within the group that improved on the GROC anchor. This finding also supports the discriminating property of the GROC-NP as an external anchor. Further support of the construct validity of the NPRS-NP (also GROC-NP) was provided by the between-group difference in the NPRS score change, between the stable and improved groups.

The results also confirmed our hypothesis regarding the concurrent validity of NPRS-NP, with NPRS change scores moderately correlated with GROC-NP (*r* = 0.43), which is within the range reported in the literature (*r*s = 0.26–0.57) [25, 28].

### Responsiveness

The NPRS-NP was found to be sensitive to change with an MIC ranging from 1.17 to 1.33 for small to large improvements. The MIC in the current study meets the requirement of being greater than the MDC value for the NPRS-NP which means that the value for important change exceeds measurement error, in contrast to the previous studies [25, 28]. The previous studies have found the MIC for NPRS between 0.9 and 4.5 [12, 13, 25, 28, 32–34, 38], with value closer to 2 as the most commonly accepted important change for the patients with both acute and chronic pain conditions [32, 33]. The MIC of NPRS-NP in the current study is comparable to the previous research by Cleland and colleagues (MIC 1.3) [25] and Mintken and colleagues (MIC 1.1) [28] which reported MIC values for neck pain and shoulder pain respectively. Higher MIC values (between 2.2 to 4.5) have also been reported in the studies on LBP [34, 38]. The range of MIC estimates across small, medium and large improvements is narrower than a previous report, which showed estimates for the NPRS of 1.5, 3.0 and 3.5, respectively [12]. Variations in the values of MIC can be a result of variations in the method of assessment of MIC [34, 39], the population sampled, and chronicity of the condition [34]. For example, van der Roer and colleagues studied MIC in sub-acute and chronic LBP and found that values of MIC were greater for chronic conditions compared to sub-acute conditions for a number of outcome measures (which also included NPRS) [34]. Finally, the findings on MIC of our study is slightly higher than MIC on children and adolescent (MIC = 0.9–1.0), as reported in a recent systematic review [13].

### Strengths and limitations

The results of the current study are supported by a strong methodology, demonstrated by; no loss to follow-up, two independent measurement points at a mean interval of 11.5 days, and the external GROC measurement confirming the stable and improved groups. However, the study also encounters a number of limitations. First, the COSMIN checklist rates the methodological quality of a study on test-retest reliability as excellent if the sample is more than 100 [40], a larger subset for each of the stable and improved groups may have strengthened the results on reliability. We recommend the use of more than a single measurement of pain intensity, including the assessment of worst pain, best pain, average pain and current pain in the clinical setting which is suggested to increase the reliability of the pain intensity assessment [6].

Second, assessment of overall change using a GROC scale is a standard practice in psychometrics study, GROC score depends on overall change and not just pain intensity. For the same reason, the COSMIN recommends to ask patients' perception of improvement on the same construct (i.e. pain intensity in this case) than their global improvement [6]. Considering this recommendation, the construct validity of NPRS-NP was not entirely met. Future research might test the psychometric properties of NPRS by utilizing two versions of GROC i.e. one that asks participants to rate their (1) global improvement and (2) specific improvement in pain intensity to see if they yield different psychometric properties of NPRS-NP.

Third, the sensitivity values of NPRS-NP ranged between 0.43 to 0.64, which indicate that the diagnostic ability of NPRS-NP to distinguish between the stable and improved group should be reconsidered. De Vet and colleagues questioned the application of MIC at individual level if the sensitivity and specificity of a measure are less than 75% [6]. The reasons for the lower values of sensitivity of the NPRS-NP may be due to the difficulty in understanding the concept of the NPRS because the sample included a large proportion of the participants with low education level and varied ethnicity. Although the number of participants who struggled to complete NPRS was not documented, it was noted that repeated explanations had to be given on the numerical nature of the NPRS-NP before some participants were able to complete the NPRS-NP scale. Other participants did not rate the pain intensity in a single number and reported their intensity of pain in a range; for example 3–5 out of 10. In these cases, we consistently recorded the higher number as the participant’s response. In contrast to the difficulties in completing the NPRS-NP, participants easily completed the GROC scale, probably due to the descriptive nature of GROC which has verbal descriptors in addition to a numeric scores. We recommend that Nepalese should be asked for their preferences for the choice of measure for assessment of pain intensity in future research to assess if they prefer other measures of pain assessment such as a verbal rating scale or a faces pain rating scales over numerical rating scale, due to this apparent difficulty with numerical rating.

Fourth, it is also worth noting that the sample used in this study comprised of a variety of ethnic groups, which could also raise a question whether differences in ethnicity may have affected the study findings. As we included only participants who could fluently speak and understand Nepali, variation in ethnicity may be unlikely to have influenced our results. Inclusion of individuals with lower education and different ethnic groups could be considered a strength of the study, as it improves the generalizability of the study findings to the Nepalese population.

Finally, as the NPRS is considered an ordinal scale, caution should be used with regard to treating it as a ratio scale like visual analogue scale (VAS); this is considered an important disadvantage of it as a measure for assessment of pain intensity in research [41]. Nevertheless, researchers have argued that an outcome measure with multiple items using a Likert scale can generally be confidently treated as an interval scale [42]. Likewise, research investigating the correlations of NPRS with VAS have consistently found strong correlations both in the adult (*rs* = 0.94–0.96) [43, 44] and pediatric populations (*rs* = 0.74–0.96) [13].

## Conclusions

The Nepali version of NPRS and GROC were successfully translated after cultural adaptations. NPRS-NP demonstrated good reliability, validity, and ability to detect change in pain intensity over time in Nepalese with musculoskeletal pain.

## Additional files

Additional file 1: The Nepali version of Numerical Pain Rating Scale (NPRS). (PDF 345 kb)

Additional file 2: The Nepali version of Global Rating of Change (GROC-NP). (PDF 342 kb)

## Abbreviations

AUC: Area under the curve
CI: Confidence interval
COSMIN: COnsensus Based Standards for the selection of health Measurement INstruments
GROC: Global rating of change
GROC-NP: Nepali version of global rating of change
ICC: Intraclass correlation coefficient
IMMPACT: Initiative on methods, measurement, and pain assessment in clinical trials
LBP: Low back pain
MDC: Minimum detectable change
MDC_90_: Minimum detectable change at 90% confidence margin
MIC: Minimum important change
NPRS: Numerical pain rating scale
NPRS-NP: Nepali version of numerical pain rating scale
PROMs: Patient-reported outcome measures
ROC: Receiver operating characteristic
SD: Standard deviation
SEM: Standard error of measurement
