# Supplementary material for: Translation, cross-cultural adaptation and psychometric properties of the Nepali versions of numerical pain rating scale and global rating of change
Source: Health Qual Life Outcomes. 2017 Dec 4;15:236. doi: 10.1186/s12955-017-0812-8 (PMC5715544; doi:10.1186/s12955-017-0812-8)
Supplement: Supplementary file 1 — The Nepali version of Numerical Pain Rating Scale (NPRS). (PDF 345 kb) [file 12955_2017_812_MOESM1_ESM.pdf]

दुखाइको तिब्रता मापन प्रश्नावली

<u>प्रारम्भिक जाँच</u> :

म तपाईको ____________ दुखाइको मात्रा सोध्न गइरहेको छु । तपाईले आफ्नो दुखाइलाई ० देखि १० सम्मको अंकमा मूल्यांकन गर्नु पर्ने हुन्छ | सुन्ना "०" भनेको "दुखेको छैन" हो भने "१०" भनेको अत्याधिक दुखाइ (अथवा सहन नसक्ने दुखाइ) हो ।

कृपया आफ्नो दुखाइको तिब्रता मूल्यांकन गर्नुहोस् ।

- "अहिले ० देखि १० मा कति दुखेको छ ?"
- "बितेको २४ घण्टाको सबै भन्दा धेरै दुखाइ कति थियो?"
- "बितेको २४ घण्टाको सबै भन्दा कम दुखाइ कति थियो?"

कुनै एक अंक भन्नुहोस् वा एक अंकमा चिन्ह लगाउनुहोस् ।

० १ २ ३ ४ ५ ६ ७ ८ ९ १०

दुखाइ छैन

अत्याधिक दुखाइ
(सहन नसक्ने दुखाइ)

	**प्रारम्भिक जाँच मिति**	**पुन जाँच मिति**	**पुन जाँच मिति**
अहिलेको दुखाइ			
बितेको २४ घण्टाको सबै भन्दा धेरै दुखाइ			
बितेको २४ घण्टाको सबै भन्दा कम दुखाइ			
**जम्मा**			

NPRS दुखाइ स्कोर
