# Supplementary material for: Translation, cross-cultural adaptation and psychometric properties of the Nepali versions of numerical pain rating scale and global rating of change
Source: Health Qual Life Outcomes. 2017 Dec 4;15:236. doi: 10.1186/s12955-017-0812-8 (PMC5715544; doi:10.1186/s12955-017-0812-8)
Supplement: Supplementary file 2 — The Nepali version of Global Rating of Change (GROC-NP). (PDF 342 kb) [file 12955_2017_812_MOESM2_ESM.pdf]

Global Rating of Change (GROC) – Nepali Version

समग्र परिवर्तन मापन प्रश्नावली

	प्रथम उपचार	दोश्रो उपचार	अन्तिम उपचार
GROC	XX	/ /	/ /

मिति : ______ / ______/ ______
दिन महिना वर्ष

तपाईंले पहिलो पटक उपचारको क्रममा आउँदा आफ्नो समस्या कसरी बयान गर्नु भएको थियो र पहिलेको तुलनामा आज कस्तो अनुभव गर्नु भएको छ ? कृपया बयान गर्नुहोस् ।
(केवल एक मात्र बाकसमा "✓" चिन्ह लगाउनुहोस्):

७ ☐ धेरै सुधार

६ ☐ मध्यम सुधार

५ ☐ थोरै सुधार

४ ☐ **पहिले जस्तै**

३ ☐ थोरै बिग्रिएको

२ ☐ मध्यम बिग्रिएको

१ ☐ धेरै बिग्रिएको

फिजियोथेरापिस्ट:

"मध्यम बिग्रिएको" अथवा **"धेरै बिग्रिएको"** बाकसमा चिन्ह लगाउनु भएमा कारण पनि उल्लेख गर्नुहोस् ।
